# Knowledge, attitude, and patient advice on sustainable diets among Spanish health professionals

**DOI:** 10.3389/fnut.2023.1182226

**Published:** 2023-07-17

**Authors:** Ujué Fresán, M. Carmen Vidal-Carou, Guadalupe Ramos-Truchero, Miguel Sáenz de Pipaon, Luis A. Moreno, Jordi Salas-Salvadó

**Affiliations:** ^1^eHealth Group, Barcelona Institute for Global Health (ISGlobal), Barcelona, Spain; ^2^Institut de Recerca en Nutrició i Seguretat Alimentària (INSA-UB) Maria Maetzu Unit of Excellence Program, Universitat de Barcelona, Santa Coloma de Gramenet, Spain; ^3^Departament de Nutrició, Ciències de l'Alimentació i Gastronomia, Campus de l'Alimentació de Torribera, Universitat de Barcelona, Santa Coloma de Gramenet, Spain; ^4^Faculty of Education and Social Work, Department of Sociology and Social Work, University of Valladolid, Valladolid, Spain; ^5^Neonatology, Instituto de Investigación Sanitaria del Hospital Universitario La Paz—IdiPAZ, (La Paz University Hospital—Universidad Autónoma de Madrid), Madrid, Spain; ^6^Consorcio CIBER, M.P. Fisiopatología de la Obesidad y Nutrición (CIBERObn), Instituto de Salud Carlos III (ISCIII), Madrid, Spain; ^7^GENUD (Growth, Exercise, Nutrition and Development) Research Group, Universidad de Zaragoza, Instituto Agroalimentario de Aragón (IA2) and Instituto de Investigación Sanitaria Aragón (IIS Aragón), Zaragoza, Spain; ^8^Departament de Bioquímica i Biotecnologia, Unitat de Nutrició Humana, Universitat Rovira i Virgili, Reus, Spain; ^9^Alimentaciò, Nutrició, Desenvolupament i Salut Mental (ANUT-DSM) Department, Institut d'Investigació Sanitària Pere Virgili (IISPV), Reus, Spain

**Keywords:** dietary survey, dietary environmental impact, food sustainability, food concerns, dietary counseling, sustainable knowledge, sustainability awareness, health personnel

## Abstract

Current dietary patterns, especially in high-income countries, are unsustainable. Health professionals, due to their credibility and close contact with the general population, could serve as agents of change for the adoption of sustainable diets. The objective of this study was to assess the knowledge and attitude regarding sustainable diets among the health professionals in Spain. A 24-item online questionnaire was designed for this purpose, and sent to health professionals (i.e., dietitians-nutritionists, nurses, physicians, and pharmacists). From September 2021 to May 2022, 2,545 health professionals answered the survey completely. One-fifth of them had never heard the term “sustainable diet”, and most of them recognized having limited knowledge about it. They considered promoting sustainable diets when making dietary recommendations important, and pointed out that they would like to be trained on the topic. Indeed, they reported that all health professionals, independent of their career background, should be educated on sustainable diets. Efforts should be stressed on implementing training courses, at university level but also as continuous post-graduate training, providing health professionals in Spain the necessary knowledge to promote the adoption of sustainable diets among the population.

## 1. Introduction

The current food system is unsustainable. While millions of people are suffering from nutrition and food insecurity (1), more than one-third of all food is lost or wasted (2); healthy diets are not affordable and accessible to a large proportion of the world's population (3), and unhealthy diets themselves are a leading cause of morbidity and mortality worldwide (4); unfair salaries and working conditions, forced and infant labor within the food system are constantly reported (5, 6), as long as inequalities between large companies and small producers and farmers persist (7); additionally, the food system is among the main drivers of environmental pollution, natural resources usage, and biodiversity loss (8). Altogether, changing the current food system toward a fair and sustainable one, that provides healthy diets for all within planetary boundaries, is timely. This transformation requires the engagement of all stakeholders, from producers to consumers (9, 10).

The Nutrition sciences need to move beyond the biological dimension, to also address ethical concerns that include social and ecological factors, that, in the end, also affect human health (11, 12). According to the FAO definition, “Sustainable diets” are those diets that are nutritionally balanced, safe and healthy, and that contribute to nutrition and food security, as well as a healthy life for present and future generations. With that aim, sustainable diets have low environmental impact and are respectful of ecosystems and biodiversity, are economically fair, affordable, accessible, and culturally acceptable (13). To encourage the adoption of sustainable diets among the population, a multilevel action approach is necessary, in which nudges and policies are combined with initiatives explicitly tailored to educate and motivate individuals toward the adoption of such diets (14). Indeed, the latest could be a short-term strategy to promote such an urgent dietary behavior change while structural changes and policies are being implemented.

Spain, like other high-income countries, has experienced a dietary transition in recent years. The traditional Spanish diet, rich in whole plant-based foods, has evolved into a diet high in animal-sourced and convenience foods, making the current diet an unhealthy pattern with high environmental impact. It has been estimated that, compared to the current average Spanish diet, the general adoption of healthy plant-based diets could prevent more than 80,000 deaths per year while reducing greenhouse gas emissions by at least 70%, and the use of natural resources such as water, soil, nitrates, and phosphates by 25–55% (15). Reducing food waste is another pending task for the Spanish population, who each Spaniard wastes 77 kg of food yearly (2). Additionally, at the socio-economic level, it has become clear that part of the food consumed in the country does not come from fair sources (6). All the above highlights that Spanish society also needs to change its eating behaviors toward a sustainable diet. The Spanish population seems to be willing to follow a sustainable diet, but they acknowledged not knowing how to follow such a diet (16).

Health professionals that provide dietary counseling, because of their credibility, close contact with the general population, and influence on their dietary choices, have been pointed to as key partners for the promotion of sustainable diets (17). Recent studies suggest, however, that health professionals are not literate on sustainable diets, and that their promotion from healthcare centers lies with their individual knowledge and beliefs (17). So far, those studies have mainly focused on professionals from US and Canada, and to a lesser extent on Europeans. No study has targeted health professionals from Spain. Thus, the main objective of this study was to evaluate the knowledge and attitude, and also the practice of recommending sustainable diets, among health professionals in Spain.

## 2. Materials and methods

An online *de novo* questionnaire was designed by experts on nutrition, communication, and sociology to conduct this study. The survey consisted of 24 multiple-choice questions and collected information on sociodemographic characteristics (sex, age), professional background (profession, years of professional experience, highest academic degree attained, frequency of taking continuous training courses, and reading of nutrition-related scientific papers), knowledge and awareness about food sustainability, and also the practice of providing dietary recommendations considering food sustainability (Supplementary Table 1, Spanish and English versions). The survey did not contain any items to capture sensitive data, nor data that could identify the participants. Thus, the study is exempt from requiring the approval of any Ethics Committee. The estimated time required to answer the survey was around 15–20 min.

Between September and December 2021, different Spanish Councils of health professional Colleges were contacted. Online meetings were organized with representatives of each of them individually, explaining the purpose of the study, and requesting their collaboration. The option of refining the questionnaire was also offered. Four of them (i.e., Consejo General de Colegios Oficiales de Médicos, Consejo General de Enfermería, and Consejo General de Colegios Farmacéuticos y Academia Española de Dietistas-Nutricionistas de España) agreed to collaborate. They sent an email to all their affiliated professionals explaining the purposes of the study, asking for participation, and providing the link to the survey. The survey, based on a computer-assisted web interview, remained open between September 2021 and May 2022.

Out of the 652,600 invited professionals (i.e., 310,000 nurses, 260,000 physicians, 76,900 pharmacists, and 5,700 nutritionists.), 4,567 of them started the questionnaire. Two thousand and twenty-two respondents were excluded for not completing the entire questionnaire. The final study population analyzed included 2,545 participants, 1,139 being nurses, 427 physicians, 346 pharmacists, 550 nutritionists-dietitians, and 83 who did not report their profession.

Descriptive statistics (percentages) were used to assess the results. The participation rate among the different groups of health professionals was not equal. As the objective of this study was not to compare different groups of health professionals but to assess the knowledge and attitude of health professionals as a whole, the results were weighted considering these differences to make the results equally representative of the different health professions. The distribution of the sample universe and number of answered questionnaires by type of health professional, as well the weight applied for the answers of each type of professional can be found in Supplementary Table 2.

## 3. Results

### 3.1. Study population

Most of the sample were women (76%) between 35 and 64 years old (78.7%). The highest level of education achieved by almost half of the sample (49.1%) was a bachelor's degree, while 39% also had a Master's degree and/or a doctorate. Roughly three out of four of the respondents (72.6%) had professional experience of more than 15 years, and only 7.7% had <5 years. Most of them (80.6%) stated they participate annually in continuing post-graduate training sessions or attend scientific conferences, although 71.1% recognized reading less than two scientific papers related to nutrition and dietetics per month (Table 1).

**Table 1:** Sociodemographic characteristics and professional trajectory of the surveyed health professionals.

	**Percentage of the sample**
**Sex**	
Woman	76.0
Man	23.2
Prefer not to disclose	0.8
**Age**	
Below 35 years old	13.7
Between 35 and 49 years old	39.3
Between 50 and 64 years old	39.4
65 years or above	7.5
**Years working as a health professional**	
5 or fewer years	7.7
Between 6 and 15 years	19.8
Between 16 and 25 years	31.1
Between 26 and 35 years	27.5
36 or more years	14.0
**Highest academic degree**	
Bachelor's degree	49.1
Postgraduate degree	11.9
Master's degree	26.5
PhD	12.5
**Frequency of participation in continuous training courses or attendance at professional or scientific conferences**	
Never	3.5
Once every 5 years	5.4
Once every 2 years	10.4
Once a year	29.5
More than once a year	51.1
**Frequency of reading nutrition-related scientific papers**	
Never	12.8
< 2 papers per month	58.3
1 or 2 papers per week	19.0
Between 3 or 5 papers per week	5.9
6 or more papers per week	4.0

### 3.2. Knowledge and attitude about sustainable diets

One-fifth (21.5%) of the health professionals had not previously heard about “sustainable diets”. Of those who had done, 44.0% had heard about it through channels unrelated to the healthcare profession, such as the press, social media, or informal conversations. Professional channels, such as dietary guidelines, scientific conferences, scientific papers, continuous education courses, or during their training as a health professional were reported by <20% of the sample (Table 2). However, it is worth highlighting that 62.9% of those younger than 25 years of age acknowledged having known about sustainable diets during their professional training (data non-shown).

**Table 2:** Knowledge and attitude about sustainable diets of the surveyed health professionals.

	**Percentage of the sample**
**Have you ever heard about the term “sustainable diet”?**	
Yes, in channels not related to my healthcare profession (i.e., press, social media, informal conversations, etc.)	44
Yes, during my training as a health professional	11.7
Yes, in scientific papers	17.2
Yes, in conferences	11.6
Yes, in postgraduate programs or continuous training courses	12.1
Yes, in dietary guidelines	15.7
No	21.5
**How relevant do you think it is for your clients, patients and society in general taking the broad concept of dietary sustainability into account in their dietary choices?**	
Very important	60.6
Important	32.3
Neither important nor not important	4.5
Of little importance	1.9
Not important at all	0.7
**Which of the following healthcare professionals do you think should be trained on the environmental impact of food and diet to translate that information when providing dietary counseling?**	
Primary care physicians	31.6
Physicians of other specialties	2.2
Primary care nurses	31.3
Nurses of other specialties	2.9
Pharmacists	5
Dietitians and Nutritionists	27.9
All of them	70.8
Neither of them	0.7
**What level of knowledge do you think your clients or patients have about the environmental impact of their diet?**	
Very high	0.5
High	2.5
Medium	17.5
Low	43.1
Very low	24
I do not know	12.4
**Which level of literacy do you feel you have about the environmental impact of diet?**	
Very high	27.9
High	37.9
Medium	26.4
Low	6
Very low	1.8
**Would you like to increase your knowledge of the environmental impact of food?**	
Yes, I consider it relevant as a health professional	60.7
Yes, I consider it relevant from a personal point of view	51.2
Yes, I consider it relevant for me as a citizen	45.9
No, it is not a relevant topic	2.8
**When making dietary recommendations, do you consider it relevant to take social impact factors related to food production into account?**	
Yes, I do	79.9
No, I do not	8.4
I do not know/I do not answer	11.7
**Which of the following health professionals do you think should be trained on the social dimension of diet for routine management of their clients or patients?**	
Primary care physicians	28.7
Physicians of other specialities	2
Primary care nurses	28.7
Nurses of other specialities	3.8
Pharmacists	6
Dietitians and Nutritionists	29
All of them	68.7
Neither of them	0.8
**Would you like to increase your knowledge of the social impact of food?**	
Yes, I consider it relevant as a health professional	60.9
Yes, I consider it relevant from a personal point of view	51.2
Yes, I consider it relevant for me as a citizen	46
No, it is not a relevant topic	2.9
**When you provide dietary recommendations, what importance do you give to each of the following dimensions? Please, share 100 points between the three possible options**.	
Human health	61.2
Environment	17.4
Socio-economical	21.4

After presenting to the participants the definition of “sustainable diet” provided by the FAO in 2010 (13), most of them considered important (32.3%), or very important (60.6%) that the population take into account all dimensions of sustainable diets (i.e., human health, environment, and socio-economical dimensions) when making food choices, although they considered human health dimension more relevant than the other two (Table 2). The consumption of foods high in sugars, highly processed, and containing heavy metals were the most concerning diet-related health effects for health professionals. To a lesser extent, they also considered of concern the consumption of foods high in fat, the presence of organic pollutants, pathogens, antibiotics, pesticides, additives, and allergens, or the consumption of transgenic foods (Supplementary Figure 1).

Two out of three (67.1%) thought that their patients had a low level of knowledge about the environmental impact of their diet. The majority of health professionals recognized that their own knowledge about the dietary environmental impact is not high: 37.9% valued their knowledge level as middling, and 32.4% said it was low or very low. Only 29.7% considered themselves to have a high or very high level of knowledge. A large proportion of them considered it relevant to broaden their knowledge of both the environmental impact of diets (60.7%) and their socio-economic aspects (60.9%). Around seven out of ten of the respondents stated that all health professionals, independently of their career background, should receive training on the impact—both environmental (70.8%) and social (68.7%)—of food and diet to transfer sustainable dietary habits to their patients (Table 2).

**Figure 1:**
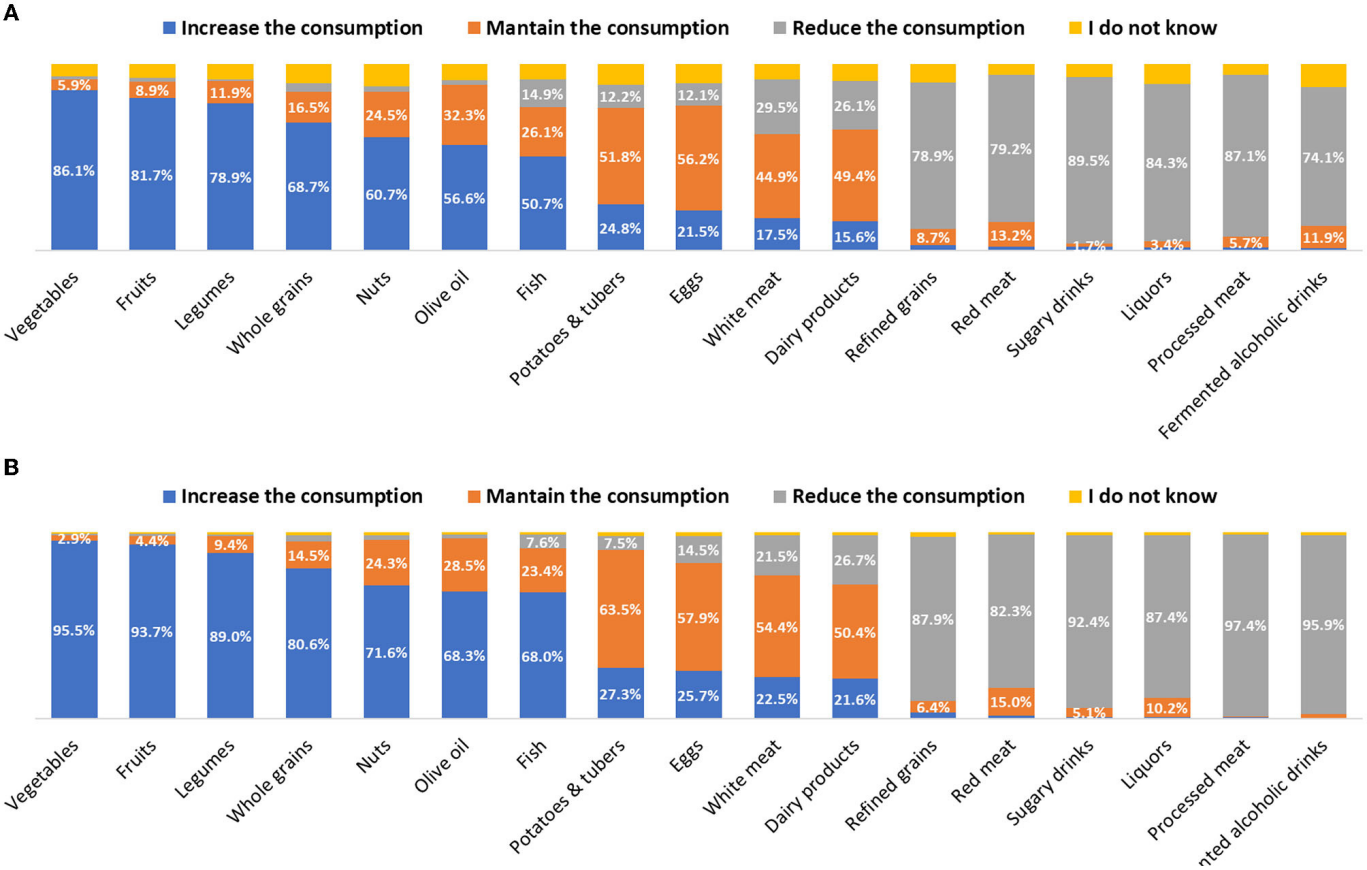
Dietary changes that the Spanish population should take for environmental **(A)** and health **(B)** reasons according to the surveyed health professionals.

When asking participants if the frequency of consumption of several food groups is adequate from health and environmental perspectives separately, they reported that Spaniards should increase their consumption of fruits, vegetables, legumes, whole grains, nuts, olive oil, and fish, and decrease their consumption of refined grains, red and processed meats, and alcoholic and sugary drinks for a healthier diet. Similar results were reported when asking about dietary changes to reduce the dietary environmental impact (Figure 1).

### 3.3. Dietary recommendations

**Figure 2:**
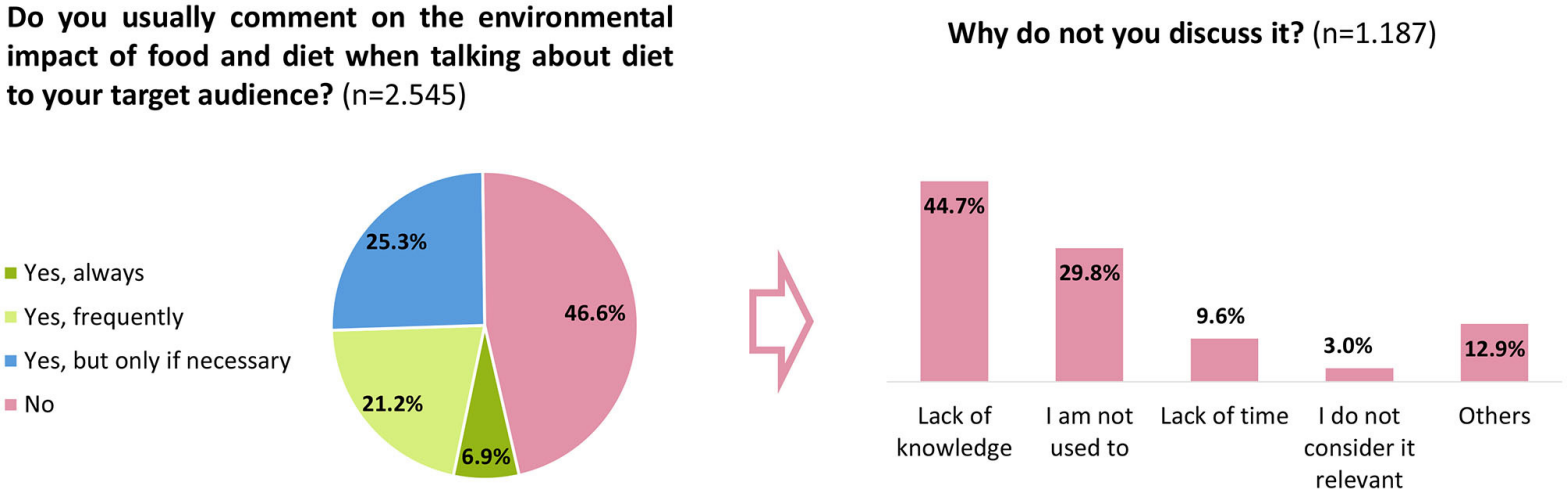
Habit of mentioning the environmental impact of food when talking about diet with health professionals' target audience.

**Figure 3:**
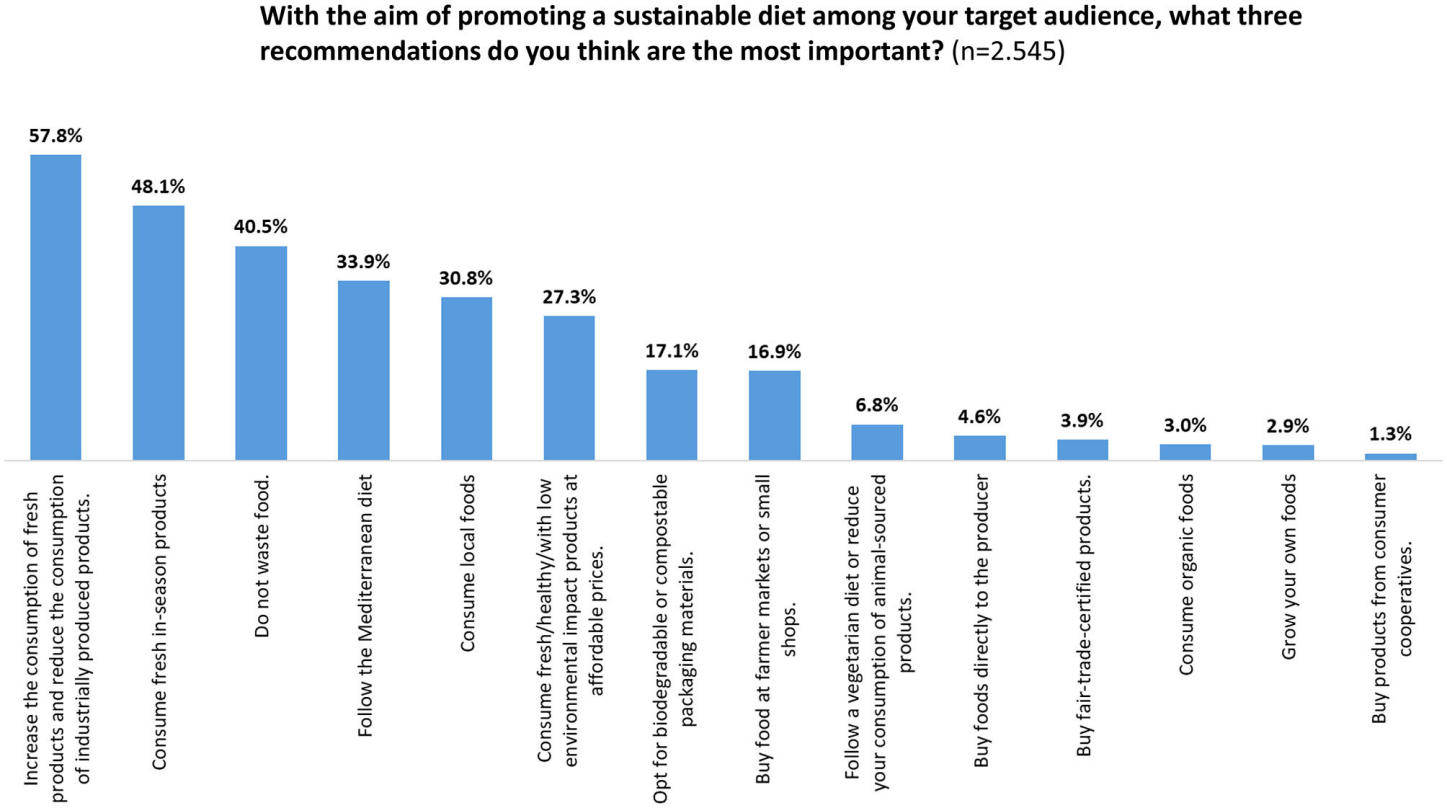
The most important recommendations to follow a sustainable diet according to the surveyed health professionals.

About half (46.6%) of the health professionals were not used to commenting on the environmental impact of diet when talking about food to their target population. This was due to a lack of knowledge (44.7%) or, to a lesser extent, because they were not in the habit of doing so (29.8%). Just 3% said that it was because they do not consider it relevant enough to be mentioned. Among those who were used to discussing this, half referred to the environmental impact of food choices often or always, and the other half occasionally (Figure 2). They expressed the view that the most relevant recommendations for following a sustainable diet would be (Figure 3): “increase the consumption of fresh food and reduce the consumption of industrial foods” (57.8%), “consume in-season fresh products” (48.1%), and “do not waste food” (40.5%). About one-third reported “follow the Mediterranean diet” (33.9%) and “consume local food” (30.8%) as the most effective strategies. They considered it less relevant to recommend “decrease the consumption of animal-sourced foods or follow a vegetarian diet” (4.6%), “buy directly from the producer” (3.9%), or “from co-ops” (1.3%), or “opt for fair-trade foods” (3.9%). Meal planning (80.6%) and the use of leftovers (72.5%) were the most important and recommendable actions to reduce food waste according to the health professionals surveyed (Supplementary Figure 2).

## 4. Discussion

This study is the first one aiming to assess the knowledge and attitudes toward sustainable diets, and the practice of recommending them, among health professionals in Spain. Our findings suggest that most of the professionals were not literate on dietary environmental and social dimensions. Nevertheless, they considered it important to promote sustainable diets when making dietary recommendations, and pointed out that all health professionals, independently of their career background, should be educated and trained on sustainable diets to provide dietary counseling considering not only on the healthiness, but also on the dietary environmental impact, social aspects, and economic considerations.

### 4.1. Ways to educate and train health professionals in sustainable diets

Our results show that, on one hand, one-fifth of the respondents recognized never having heard the term “sustainable diets” before, and on the other, that channels related to the healthcare profession (such as dietary guidelines, scientific conferences, continuous training programs, or during their formal education as a health professional) were not the main channel through which healthcare professionals were made aware about this topic. This fact points out the need in Spain to evolve promoting, educating, and training on nutrition and dietetics from a simplistic biological vision to a broader one.

Dietary guidelines, which are the basis for national food policies and public institutions' feeding programs, are one of the most relevant areas to leverage not only a large-scale adoption of sustainable diets but also literacy on nutrition and dietetics. Until now, environmental sustainability had not been a recurrent feature in dietary guidelines. Some even, such as the 2015–2020 Dietary Guidelines for Americans, stated that dietary recommendations should be based exclusively on the healthiness of food, showing a clear refusal to consider environmental sustainability aspects (18). Fortunately, things are changing; including in Spain. The Spanish Agency for Food Safety and Nutrition has very recently released new dietary guidelines not only considering the healthiness but the overall sustainability of dietary patterns (19). It should be noticed that our survey took place before the release of the new guidelines. By now, the new dietary recommendations may have reached a large number of health professionals. If the survey were to take place now, it is possible that the proportion of health professionals who report not having heard about sustainable diets would be lower, and the percentage of professionals aware of them through dietary guidelines higher.

On the other hand, while health professionals reported attending professional and scientific conferences or taking continuous training courses often, only around 12% of our sample population indicated having heard about sustainable diets in conferences or courses. This is definitely a missed opportunity. More efforts should be made into the design and implementation of continuous training courses, and professional and scientific conferences, approaching a holistic view of diets. These would be a golden opportunity to disseminate about dietary sustainability, providing knowledge and tools to health professionals for encouraging the adoption of sustainable diets among the general population (20).

Of course, universities should expedite the incorporation of sustainable diets in their curriculum to also educate and train future health professionals that are potential key actors to sensitize the general population; our findings pointed out that still more than one-third of health professionals younger than 25 years have not been trained on sustainable diets during their professional training. Other educational formats, such as topic-oriented courses or workshops, could also be good resources for educating on sustainable diets from universities (20). Residency or professional practice programs should complement the theoretical knowledge acquired through the curriculum, as these periods would provide an opportunity for young professionals to acquire practical training to be applied later during their careers (20).

### 4.2. Health professionals' knowledge of sustainable diets comes from sources unrelated to their professional training

Health professionals recognized reducing food waste and buying local food among the most relevant actions to promote a sustainable diet, while buying directly from the producers or in cooperatives, or reducing the consumption of animal-based products were placed among the less relevant ones. It should be noticed, however, that the questionnaire only allows participants to select three options as the most relevant ones. It is possible that participants also consider those less popular options important, although to a lesser extent. These findings could be interpreted as a sign that the press and social media are their main sources of information, as they acknowledged.

Large campaigns focused on the promotion of local foods, including meats and dairy products, and the reduction of food waste has been launched during the last years in Spain (21, 22). In accordance, the general population—including health professionals, as our findings indicate—have got the idea that giving priority to local food and reducing food waste, are the most relevant strategies to advance toward a sustainable diet, while the high environmental impact of animal-based foods is not well-perceived (16). However, although it is true that tackling food waste is key in the transition toward a sustainable diet, people should be aware that not all local products are healthy, or socially or environmentally sustainable options (23), and that reducing the consumption of animal-based food is one of the best measures to reduce the dietary environmental impact (9). On the other hand, opting for buying food directly from producers or from cooperatives is a way of reconnecting with the production areas and their actors (24), strengthening the relations between consumers and producers toward a more equitable, and fairer food system (25), and supporting the maintenance of the job of those small producers, to stand up to the pressure of big food industries (7).

Massive campaigns have the power of disseminating knowledge and motivating receptors to change their behaviors. Honest campaigns promoting the adoption of healthy, environmentally sustainable, and socio-economically fair diets, and giving tips to follow such a diet, are urgently needed. Additionally, stricter regulation is needed in food industry advertising, to make sure that their message is aligned with public health and sustainability goals. Other public health strategies, such as food labeling, could be another soft policy measure to leverage knowledge regarding food sustainability among the general population (26).

### 4.3. Health professionals do not recognize tradeoffs between healthiness and the environmental impact of foods

The majority of the health professionals recognized having a medium or low level of knowledge on the environmental impact of food, being this the main barrier to discussing the environmental impact of food when providing dietary counseling. This lack of knowledge was evident when asking participants which changes the Spanish population would have to make to reduce their dietary environmental impact.

It seems that health professionals associate the effects of foods on the environment with those on human health. However, both dimensions are not necessarily aligned. For instance, participants responded that the population should increase their consumption of whole grains, while reducing the consumption of their refined version, because of environmental and health reasons. Although this replacement would provide health benefits, the environmental impact of both whole and refined grains is low (27). Thus, the reduction of refined grains makes sense for health reasons, but not for their environmental impact. The same happens with sugary drinks; although participants responded that their consumption should decrease for both environmental and health reasons, the fact is that their environmental impact is low (27). The case of dairy products is also striking. Besides the necessity of limiting the consumption of dairy products due to environmental reasons (28, 29), just a fourth of the sample indicated that their consumption should be reduced, and 15% even mentioned that should increase. Comparing these findings with those of a recent study aimed at the knowledge and attitude toward sustainable diets in the Spanish adult population (16), it seems that the knowledge of health professionals about the environmental impact of foods is similar to that of the general Spanish population. They also described a general association between healthiness and environmental sustainability, and a lack of awareness about the high environmental impact of dairy products, as we detected.

### 4.4. Health professionals prioritize human health effects over other sustainability dimensions

Besides most of the health professionals indicated that it is important to consider the environmental impact and socio-economic aspects when making dietary recommendations, they noted that the health dimension—both nutritional adequacy and safety—is much more relevant than the other aspects. This greater priority given to human health was shown by asking participants about the three most relevant measures to promote a sustainable diet. The recommendation they recognized as the most relevant was related to consuming more fresh products, which could be understood as a bias in favor of the health dimension. Fresh products may have been interpreted as healthy products such as vegetables and fruits, as seems to be understood by the Spanish population (16). This could be directly linked to the health dimension, but is not relevant from an environmental or socio-economical point of view. With regard to the environmental dimension, food processing is responsible for <5% of the greenhouse gas emissions derived from the food system (30) and even contributes to the reduction of food loss and waste. Most of the products that do not meet the standard “cosmetic” characteristics for being introduced in the market as fresh products are processed into preserved foods, minimizing their loss in fields (31). Additionally, waste from processed fruits and vegetables is lower than that of fresh fruits and vegetables (32). In parallel, a share of farmers' livelihoods and incomes depends on processed forms of fruits and vegetables, and they also contribute to food safety, security, and nutrition thanks to their longer shelf-life and year-round availability (31). Furthermore, it should be noticed that not all fresh products are healthy or environmentally sustainable options. For instance, high consumption of fresh red meat would lead to health problems while having a high environmental impact (27).

By definition, all dimensions should be of equal importance, as the ultimate goal of sustainable diets—i.e., contributing to nutrition and food security, and a healthy life for present and future generations—could not be achieved if not taking all dimensions into account (13). Indeed, back in 2008, the United Nations World Health Assembly already made a multilateral commitment to protect the health of the population from the fatal consequences of climate change (33). Climate change is increasingly undermining every pillar of good health, affecting physical and mental health directly and indirectly: threatening global food security, intensifying water scarcity, exacerbating the risk of infectious disease outbreaks, and negatively affecting livelihoods and the socioeconomic conditions on which physical and mental health rely on, among other effects (28). On the other hand, the direct link between socioeconomic aspects and health is well known (34). Altogether, health professionals should be aware that environmental and socio-economic problems directly and indirectly affect people's health, and should grant those dimensions the relevance they deserve when providing dietary counseling. A recent pilot study suggested that making the direct link between the environment and human health clear to health professionals could motivate them to engage in the promotion of sustainability actions (35). Presenting this narrow connection between food-health-environment-socioeconomic aspects when educating health professionals on sustainable diets could be an effective strategy for their commitment on their promotion.

### 4.5. Strengths and limitations

The major strength of this study is the large sample size, and the inclusion of health professionals from different areas, such as Nutrition and Dietetics, Medicine, Nursing, and Pharmacy. Some limitations should also be recognized. A selection and participation bias cannot be discarded; it is possible that those professionals more concerned about the topic were those who participated in the study, or even that the respondents were not as interested in sustainable diets as they reported. On the other hand, this would also indicate that the low level of knowledge detected in this study would be even more prominent. The interpretation of the results of the present study has to be done with caution. While the questionnaire was designed and externally checked by experts on nutrition, sociology, and communication, it has not received proper validation, and would be susceptible to improvements. Additionally, it has not been designed to deeply assess the knowledge of health professionals but to have a broad perspective.

## 5. Conclusions

This study, aimed at the assessment of the knowledge and attitudes toward sustainable diets among health professionals in Spain, shows that their knowledge in this area is not very high. Nevertheless, they would like to receive education and training to promote sustainable diets among their patients/clients. Due to the urgency of the general adoption of sustainable diets, the key role of health professionals in such a dietary transition, and their willingness to promote sustainable diets in their daily practices, efforts should be stressed on implementing specific guidelines for these health professionals, but also training courses, at university level but also as continuous practical training, providing to them the necessary tools to be agents of change in the general adoption of sustainable diets.

## Data availability statement

The original contributions presented in the study are included in the article/Supplementary material, further inquiries can be directed to the corresponding authors.

## Ethics statement

Ethical review and approval was not required for the study on human participants in accordance with the local legislation and institutional requirements. Written informed consent for participation was not required for this study in accordance with the national legislation and the institutional requirements.

## Author contributions

UF, MV-C, and JS-S participated in the online meeting with the representatives of the councils of health professional colleges. UF wrote the first draft. All authors contributed to the design of the questionnaire, participated in the interpretation of the data, reviewed the draft, and have read and approved the final version of the manuscript.
