# Supplementary material for: Knowledge, attitude, and patient advice on sustainable diets among Spanish health professionals
Source: Front Nutr. 2023 Jul 17;10:1182226. doi: 10.3389/fnut.2023.1182226 (PMC10389708; doi:10.3389/fnut.2023.1182226)

**Supplemental Table 1:** Survey used in the study (Spanish and English versions)

**ENCUESTA SOBRE SOSTENIBILIDAD ALIMENTARIA EN PROFESIONALES SANITARIOS: CONOCIMIENTO, GRADO DE CONCIENCIACIÓN Y RECOMENDACIONES DIETÉTICAS**

**VARIABLES DESCRIPTIVAS**

1. Antes de nada, indíquenos por favor su profesión.

2. Sexo

Hombre

Mujer

Prefiero no decirlo

3. Edad

Menor de 25 años

Entre 25 y 34 años

Entre 35 y 49 años

Entre 50 y 64 años

Entre 65 y 69 años

70 años o más.

4. ¿Cuántos años lleva ejerciendo como profesional sanitario?

Menos de 5 años

Entre 6 y 15 años

Entre 16 y 25 años

Entre 26 y 35 años

36 años o más

5. ¿Cuál es su máximo nivel académico alcanzado?

Diplomatura/Licenciatura/Grado

Postgrado

Máster

Doctorado

6. Indique, por favor, con qué frecuencia participa en acciones de formación continuada o asiste a congresos/conferencias científicas o profesionales.

Nunca

Cada 5 años aproximadamente

Cada 2 años aproximadamente

Cada año

Más de una vez al año

7. Indique, por favor, con qué frecuencia lee artículos científicos relacionados con nutrición y dietética.

Nunca

Menos de 2 artículos al mes

Entre 1 y 2 artículos por semana

Entre 3 y 5 artículos por semana

6 o más artículos por semana

**NIVEL DE CONOCIMIENTO Y CONCIENCIACIÓN SOBRE DIETAS SOSTENIBLES**

8. ¿Ha oído alguna vez hablar del concepto de alimentación/dieta sostenible? (Responda un máximo de tres opciones).

Sí, en cursos de postgrado o formación continuada.

Sí, en artículos científicos.

Sí, durante mi formación como profesional sanitario.

Sí, en canales y medios no vinculados a mi profesión sanitaria (prensa, redes sociales, conversaciones informales, etc.).

Sí, en congresos y conferencias.

Sí, en guías alimentaria.

No, nunca he oído hablar de dietas sostenibles.

De cara a continuar alineados durante el resto del cuestionario, le facilitamos una definición de consenso de Dieta Sostenible acuñada por la Organización de las Naciones Unidas para la Alimentación y la Agricultura (FAO): “Las dietas sostenibles son aquellas que generan un impacto ambiental reducido y que contribuyen a la seguridad alimentaria y nutricional y a que las generaciones actuales y futuras lleven una vida saludable. Además, protegen y respetan la biodiversidad y los ecosistemas, son culturalmente aceptables, accesibles, económicamente justas y asequibles y nutricionalmente adecuadas, inocuas y saludables, y optimizan los recursos naturales y humanos.”

9. ¿Cómo de relevante le parece que sus clientes, pacientes y la sociedad en general tengan en cuenta estas consideraciones propias de una dieta sostenible a la hora de elegir su alimentación?

Muy importante

Importante

Ni importante, ni poco importante

Poco Importante

Nada importante

**IMPACTO AMBIENTAL**

10. ¿Cuáles de los siguientes profesionales sanitarios cree que deberían recibir formación sobre el impacto medioambiental de los alimentos y la dieta de cara a la gestión diaria de sus clientes o pacientes? (Indique un máximo de tres, los más importantes en su opinión).

Médicos de atención primaria

Otras especialidades médicas

Enfermería de atención primaria

Otras especialidades de enfermería

Farmacéuticos

Dietistas-Nutricionistas

Todos los profesionales sanitarios anteriores

Ninguno de los anteriores

11. ¿Suele hacer mención al impacto medioambiental de los alimentos o de la dieta al hablar sobre alimentación a su público diana?

Sí, siempre

Sí, a menudo

Sí, aunque en ocasiones contadas si es necesario

No

12. Si no suele hacer mención, ¿por qué motivo?

No lo considero importante

Por falta de conocimientos sobre el tema

Por falta de tiempo

Por inercia, no tengo la costumbre

Otro (especifique)

13. Según su percepción, ¿qué nivel de conocimiento siente que tienen sus clientes o pacientes sobre el impacto ambiental de la dieta?

Muy alto, están claramente concienciados.

Alto, están bastante concienciados.

Ni alto, ni bajo.

Bajo, están poco concienciados.

Muy bajo, no están nada concienciados.

No sé, lo desconozco.

14. ¿Qué nivel de conocimiento considera que tiene usted sobre el impacto medioambiental de la dieta?

Muy alto, me considero un experto.

Alto, tengo conocimientos sobre el tema.

Ni alto, ni bajo.

Bajo, tengo poco conocimiento sobre el tema.

Muy bajo, apenas tengo conocimiento alguno.

15. ¿Le gustaría tener un mayor conocimiento sobre este concepto? (Indique todas aquellas que correspondan).

Sí, me parece que es importante para mi práctica profesional.

Sí, me parece que es interesante desde un punto de vista personal.

Sí, me parece que es interesante como ciudadano.

No, no es un concepto que me resulte interesante ni importante para mi práctica profesional.

**IMPACTO SOCIAL**

16. A la hora de realizar recomendaciones dietéticas, ¿considera relevante tener en cuenta factores de impacto social relacionados con la producción de los alimentos? (Por ejemplo, el precio, las condiciones de los trabajadores que los produjeron, o favorecer la producción familiar frente a la agroindustrial).

Sí

No

No sabe/no contesta

17. ¿Cuáles de los siguientes profesionales sanitarios cree que deberían recibir formación sobre la dimensión social de la alimentación de cara a la gestión diaria de sus clientes o pacientes? (Indique un máximo de tres, los más relevantes según su opinión).

Médicos de atención primaria.

Otras especialidades médicas.

Enfermería de atención primaria.

Otras especialidades de enfermería.

Farmacéuticos.

Dietistas-Nutricionistas.

Todos los profesionales sanitarios anteriores.

Ninguno de los anteriores.

18. ¿Le gustaría tener un mayor conocimiento sobre este concepto? (Indique todas aquellas respuestas que correspondan).

Sí, me parece que es importante para mi práctica profesional.

Sí, me parece que es interesante desde un punto de vista personal.

Sí, me parece que es interesante como ciudadano.

No, no es un concepto que me resulte interesante ni importante para mi práctica profesional.

**RECOMENDACIONES DIETÉTICAS**

19. Al proporcionar recomendaciones dietéticas, ¿qué importancia cree que se debe conceder a cada una de las siguientes dimensiones? Reparta, por favor, 100 puntos entre las tres opciones posibles.

Efectos en la salud

Impacto ambiental

Dimensión socio-económica

20. Con el objetivo en mente de promover una dieta sostenible entre sus clientes o pacientes, de las siguientes recomendaciones, indique las tres que, a su juicio, son más importantes dar.

Cultivar/producir sus propios alimentos.

Seguir la Dieta Mediterránea.

Comprar los alimentos directamente del productor.

Consumir productos procedentes de comercio justo.

Seguir una dieta vegetariana o reducir el consumo de productos de origen animal.

No desperdiciar comida.

Aumentar el consumo de productos frescos y reducir el consumo de productos de origen industrial.

Consumir productos frescos de temporada.

Comprar en el mercado local o pequeños comercios de barrio.

Adquirir los productos en cooperativas de consumidores.

Consumir productos de Km.0 o de proximidad.

Priorizar materiales de envasado biodegradable o compostable.

Consumir productos ecológicos.

Consumir productos frescos/saludables/ecológicos a precios asequibles.

21. ¿Cuáles de estas acciones considera más importantes para reducir el desperdicio de alimentos, y por tanto se necesita recomendar a la población? (Indique las dos más importantes)

Comprar menores cantidades de alimentos.

Comprar/cocinar grandes cantidades de alimentos para congelar y comer a lo largo de la semana.

Reaprovechar las sobras (cocina de aprovechamiento).

Planificar las comidas haciendo listas de compra y menús semanales.

Llevarse los alimentos sobrantes a casa tras ir al restaurante.

Separar los alimentos sobrantes para que sean utilizados como compost.

22. Indique en una escala de 1 (nada preocupante) a 5 (muy preocupante), por favor, el nivel de preocupación que le genera cada uno de los siguientes aspectos de los alimentos desde el punto de vista de su impacto en la salud. *(Opciones de respuesta: 1. Nada preocupante; 2. Poco preocupante; 3. Algo preocupante; 4. Bastante preocupante; 5. Muy preocupante; 6. No sabe/no contesta).*

Presencia de restos de plaguicidas en los alimentos.

Presencia de microorganismos patógenos.

Presencia de alérgenos (frutos secos, gluten, soja, trigo, leche, huevos).

Existencia de contaminantes orgánicos permanentes en el medioambiente como dioxinas.

Elevado contenido en azúcares.

Presencia de restos de antibióticos en los alimentos.

Alta concentración de metales pesados como mercurio, plomo o cadmio.

Uso de aditivos como colorantes, aromatizantes y conservantes.

Productos modificados genéticamente (transgénicos).

Elevado contenido en grasa.

Enfermedades transmitidas por animales (vacas locas/BSE, fiebre porcina, gripe aviar).

Alimentos altamente procesados.

23. Señale, por favor, con qué frecuencia considera usted que la población debería consumir los siguientes alimentos, en función de SU IMPACTO AMBIENTAL; es decir, del impacto que está produciendo la producción y consumo de estos alimentos en el medioambiente *(Opciones de respuesta: 1. Más de lo que se consume hoy en día; 2. En la misma proporción de lo que se consume hoy en día; 3. Menos de lo que se consume hoy en día; 4. Desconozco los efectos medioambientales de este alimento)*

Carne blanca

Pescado

Bebidas azucaradas

Aceite de oliva

Derivados cárnicos procesados (tanto de carne blanca como roja)

Patatas y otros tubérculos

Huevos

Frutos secos

Bebidas alcohólicas fermentadas (vino, cerveza...)

Lácteos

Frutas

Legumbres

Cereales refinados

Verduras

Carne roja

Bebidas alcohólicas destiladas (ron, ginebra...)

24. Por último, señale, por favor, con qué frecuencia considera usted que la población debería consumir los siguientes alimentos, por el EFECTO EN LA SALUD de cada uno de ellos *(Opciones de respuesta: 1. Más de lo que se consume hoy en día; 2. En la misma proporción de lo que se consume hoy en día; 3. Menos de lo que se consume hoy en día; 4. Desconozco los efectos en la salud de este alimento)*

Carne blanca

Pescado

Bebidas azucaradas

Aceite de oliva

Derivados cárnicos procesados (tanto de carne blanca como roja)

Patatas y otros tubérculos

Huevos

Frutos secos

Bebidas alcohólicas fermentadas (vino, cerveza...)

Lácteos

Frutas

Legumbres

Cereales refinados

Verduras

Carne roja

Bebidas alcohólicas destiladas (ron, ginebra...)

**SURVEY ON FOOD SUSTAINABILITY AMONG HEALTH PROFESSIONALS: KNOWLEDGE, ATTITUDE AND DIETARY RECOMENDATIONS**

**DESCRIPTIVE VARIABLES**

1. Kindly state your profession/occupation.

2. Gender

Male

Female

I prefer not to disclose

3. Age

Below 25 years old

Between 25 and 34 years old

Between 35 and 49 years old

Between 50 and 64 years old

Between 65 and 69 years old

70 years or above

4. How many years have you been serving as a health professional?

Less than 5 years

Between 6 and 15 years

Between 16 and 25 years

Between 26 and 35 years

36 or more years

5. What is the highest academic degree you have achieved?

Bachelor’s degree

Postgraduate degree

Master's degree

Doctorate

6. How often do you participate in continuous training programs, or attend scientific or professional conferences?

I do not participate in continuous training programs nor attend conferences.

Approximately once every 5 years.

Approximately once every 2 years.

Approximately once every year.

More than once a year.

7. How often do you read scientific papers related to the area of nutrition and dietetics?

I do not read scientific papers related to nutrition and dietetics.

Less than 2 papers per month.

Between 1 and 2 papers per week.

Between 3 and 5 papers per week.

6 or more papers per week.

**LEVEL OF KNOWLEDGE AND ATTITUDE TOWARDS SUSTAINABLE DIETS**

8. Have you ever heard about the concept of sustainable diets? (Please, select a maximum of three options).

Yes, in postgraduate or continuing training courses.

Yes, in scientific publications.

Yes, during my training as a health professional.

Yes, in channels and media not related to my health profession (press, social media, informal conversations, etc.).

Yes, at conferences.

Yes, in dietary guidelines.

No, I have never heard about sustainable diets.

In order to remain aligned for the rest of the survey, here you may find the definition of Sustainable Diet as coined by the Food and Agriculture Organization of the United Nations (FAO): "Sustainable diets are those that have a reduced environmental impact and contribute to food and nutrition security and a healthy life for current and future generations. They protect and respect biodiversity and ecosystems, are culturally acceptable, accessible, economically just and affordable, nutritionally adequate, safe and healthy, and optimize natural and human resources."

9. How relevant do you think it is for your clients, patients and society in general taking the broad concept of sustainable diets into account in their dietary choices?

Very important

Important

Neither important nor unimportant

Not very important

Not important at all

**ENVIRONMENTAL IMPACT**

10. Which of the following healthcare professionals do you think should be trained on the environmental impact of diet for routine management of their clients or patients? (Please, select a maximum of three options, the most important in your opinion).

Primary care physicians

Other medical specialties

Primary care nurses

Other nursing specialties

Pharmacists

Dietitians - Nutritionists

All of the above health professionals

None of the above health professional

11. Do you usually comment on the environmental impact of food and diet when talking about diet to your target audience?

Yes, always.

Yes, often.

Yes, but in few occasions; just if necessary.

No, never.

12. If not, why?

I do not consider it relevant.

Lack of knowledge.

Lack of time.

Inertia, I am not used to discussing it.

Other (please specify)

13. According to your perception, what level of knowledge do you feel your clients or patients have about the environmental impact of the diet?

Very high, they are clearly aware about it.

High, they are aware about it.

Medium, neither high, nor low.

Low, not very aware about it.

Very low, not aware about it at all.

I do not know.

14. How literate do you think you are about the environmental impact of diet?

Very high level of literacy, I consider myself an expert.

High level of literacy, I am knowledgeable.

Neither high, nor low level of literacy.

Low level of literacy, I have little knowledge on the topic.

Very low level literacy, I hardly have any knowledge on the topic.

15. Would you like to increase your knowledge about this concept? (Please, select all the answers that apply).

Yes, I consider it relevant for my professional practice.

Yes, I consider it relevant from a personal point of view.

Yes, I consider it relevant for me as a citizen

No, it is not a concept that I find interesting or important for my professional practice.

**SOCIAL IMPACT**

16. When making dietary recommendations, do you consider it relevant to take social aspects related to food production into account (e.g. price, working conditions, or support small-scale producers vs industrial production)?

Yes, I do.

No, I do not.

I do not know/I do not answer.

17. Which of the following health professionals do you think should be trained on the social dimension of diet for routine management of their clients or patients? (Please, select a maximum of three, the most important in your opinion).

Primary care physicians.

Other medical specialties.

Primary care nurses.

Other nursing specialties.

Pharmacists.

Dieticians – Nutritionists.

All of the above health professionals.

None of the above health professionals.

18. Would you like to increase your knowledge about this concept (please, select all that apply)?

Yes, I think it is important for my professional practice.

Yes, I find it interesting from a personal point of view.

Yes, I find it interesting as a citizen.

No, it is not a concept that I find interesting or important for my professional practice.

**DIETARY RECOMMENDATIONS**

19. When providing dietary recommendations, how important do you consider each of the following dimensions are? Please, share 100 points among the three possible options.

Healthiness.

Environmental impact.

Socio-economic dimension.

20. Please, select the three of the following answers that, in your opinion, are the most important to offer to your clients or patients to promote a sustainable diet.

Grow your own food.

Follow the Mediterranean Diet.

Buy foods directly from the producer.

Buy fair-trade-certified products.

Follow a vegetarian diet or reduce your consumption of animal-sourced products.

Do not waste food.

Increase the consumption of fresh products and reduce the consumption of industrially produced products.

Consume in-season fresh products.

Buy food at farmer markets or small shops.

Buy products from consumer cooperatives.

Buy local foods.

Opt for biodegradable or compostable packaging materials.

Consume organic products.

Consume fresh/healthy/with low environmental impact products at affordable prices.

21. Which of the following actions do you consider the most important to reduce food waste, and therefore need to be specially recommended to the population? (Please, select the two most important answers in your opinion)

Buy foods in small quantities.

Buy/cook large quantities of food to freeze and eat later on throughout the week.

Re-use leftovers.

Plan meals by making shopping lists and weekly menus.

Take home the leftovers you may have from restaurants.

Separate leftovers to be composted.

22. Please indicate on a scale from 1 (not concerning at all) to 5 (very concerning) the level of concern generated by each of the following aspects according to their impact on health *(Answer options: 1. Not* concerning *at all; 2. A little bit* concerning*; 3. Somehow* concerning*; 4. Quite* concerning*; 5. Very* concerning*; 6. I do not know/I do not answer)*

Pesticides traces.

Presence of pathogenic microorganisms.

Presence of allergens (nuts, gluten, soya, wheat, milk, eggs).

Presence of persistent organic pollutants in the environment, such as dioxins.

High sugar content.

Antibiotic traces.

High concentration of heavy metals such as mercury, lead or cadmium.

Use of additives such as colorings, flavorings and preservatives.

Genetically modified products (GM).

High fat content.

Animal-borne diseases (mad cow/BSE, swine fever, avian influenza).

Highly processed foods.

23. Please, indicate how often the population should consume the following food groups, based on their ENVIRONMENTAL IMPACT *(Answer options: 1. More than what is consumed today; 2. As much as it is consumed today; 3. Less than what is consumed today; 4. I do not know the environmental impact of this food group).*

Nuts.

Dairy products.

Sugary drinks.

Potatoes and other root vegetables.

Red meat.

Eggs.

Olive oil.

Fish.

Fermented alcoholic beverages (beer, wine, etc.).

Refined cereals.

Vegetables.

Processed meat products (both white and red processed meats).

Fruits.

Whole grains.

White meat.

Beans and legumes.

Spirits/liquors.

24. Finally, please, indicate how often the population should consume the following food groups, based on their HEALTHINESS *(Answer options: 1. More than what is consumed today; 2. As much as it is consumed today; 3. Less than what is consumed today; 4. I do not know the healthiness of this food group)*.

Nuts.

Dairy products.

Sugary drinks.

Potatoes and other root vegetables.

Red meat.

Eggs.

Olive oil.

Fish.

Fermented alcoholic beverages (beer, wine, etc.).

Refined cereals.

Vegetables.

Processed meat products (both white and red processed meats).

Fruits.

Whole grains.

White meat.

Beans and legumes.

Spirits/liquors.

**Supplemental Table 2**: Proportional distribution of the universe (N) and the sample (n) by profession, and weighting factors applied in each of them

| **Profession** | **N** | **% N** | **n** | **% n** | **Weighting factor** |
| --- | --- | --- | --- | --- | --- |
| Nurses | 310000 | 47.5 | 1139 | 46.30 | 1.0268 |
| Pharmacists | 76900 | 11.8 | 346 | 14.10 | 0.8385 |
| Phycisians | 260000 | 39.8 | 427 | 17.30 | 2.2971 |
| Dietitians-Nutritionists | 5700 | 0.9 | 550 | 22.30 | 0.0391 |
| No reported |  |  | 83 |  |  |
| Total | 652600 | 1.0 | 2462 | 100 |  |

**Supplemental Figure 1**: Level of concern for each of the following aspects of food according to their health effects


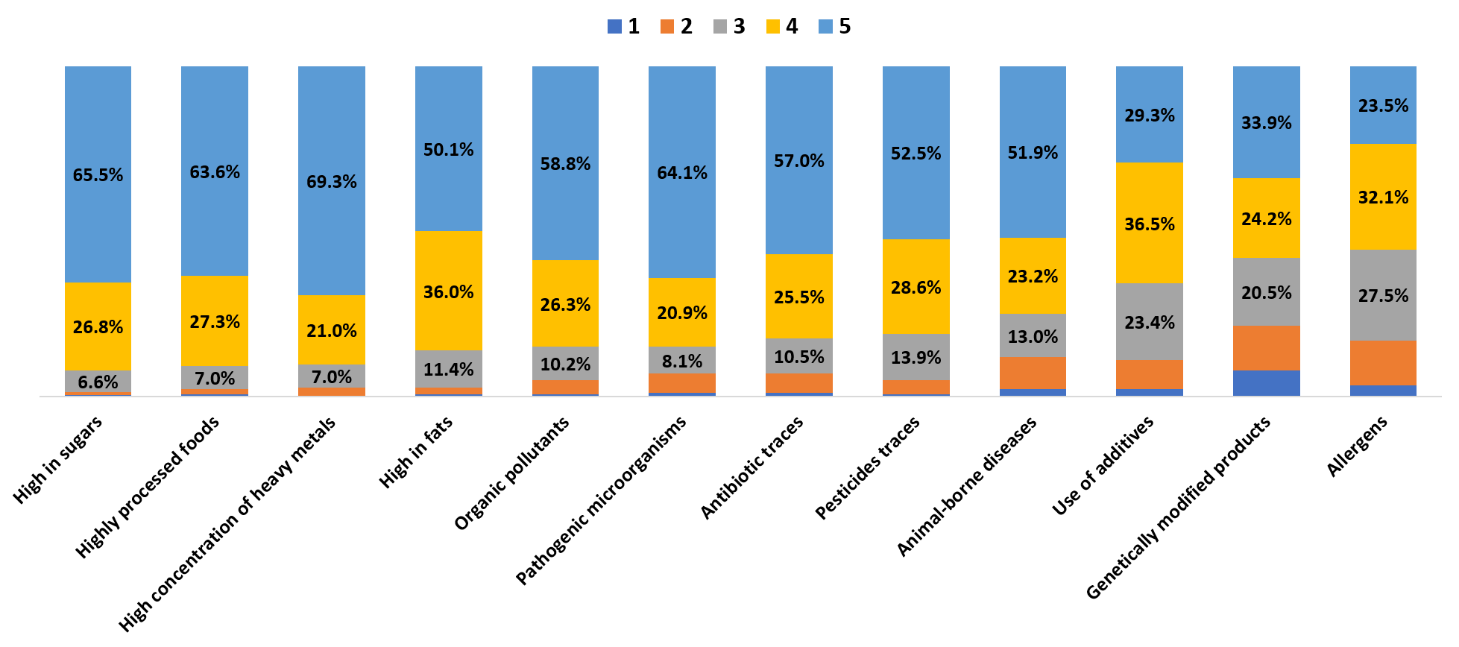


Likert scale where 1 means “no concerning at all”, and 5 “very concerning”

**Supplemental Figure 2**. The most important recommendation to reduce food waste according to the surveyed health professionals.


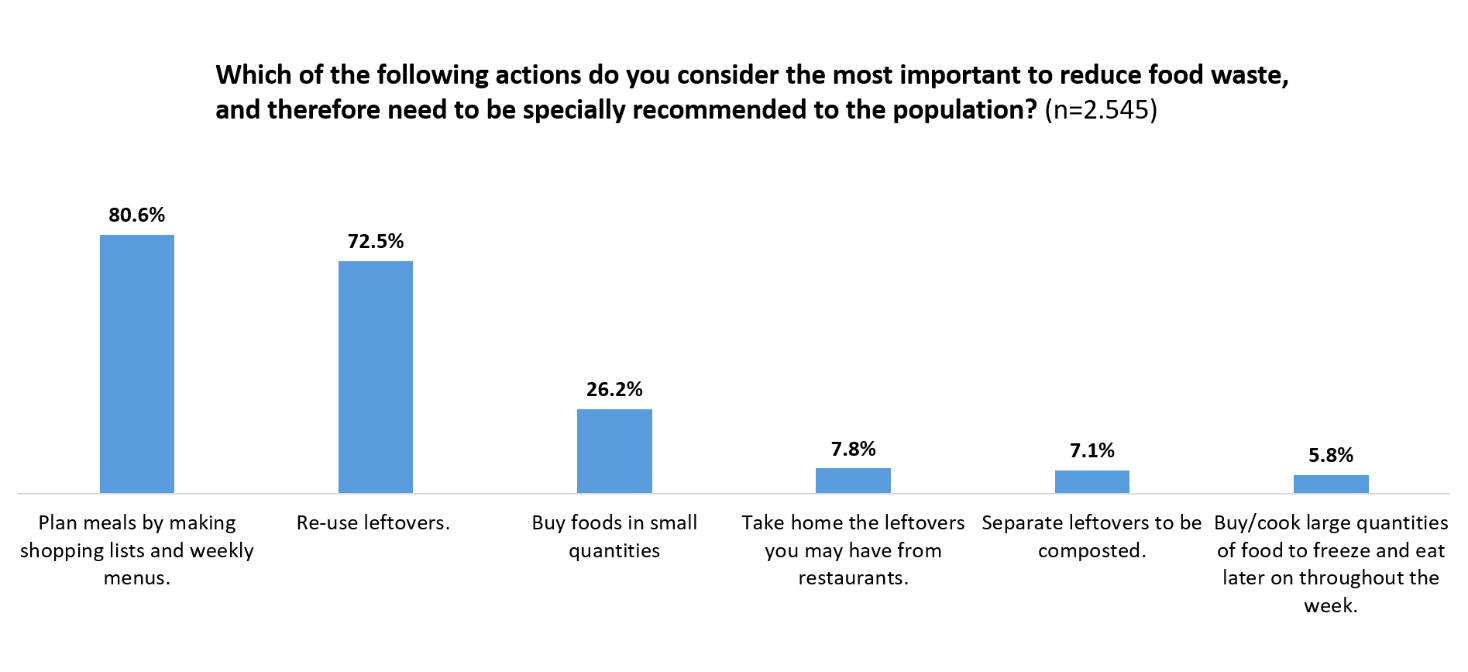

Supplement: Supplementary file 1 [file Data_Sheet_1.docx]
